# Cytoprotective Effects of Water Soluble Dihydropyrimidinthione Derivative Against UV-B Induced Human Corneal Epithelial Cell Photodamage

**DOI:** 10.3389/fphar.2021.732833

**Published:** 2021-10-20

**Authors:** Enming Du, Guojuan Pu, Siyu He, Fangyuan Qin, Yange Wang, Gang Wang, Zongming Song, Junjie Zhang, Ye Tao

**Affiliations:** ^1^ Henan Eye Institute, Henan Eye Hospital, People’s Hospital of Zhengzhou University, Henan University School of Medicine, Henan Provincial People’s Hospital, Zhengzhou, China; ^2^ Lab of Visual Cell Differentiation and Modulation, Basic Medical College, Zhengzhou University, Zhengzhou, China

**Keywords:** dihydropyrimidinthione, UV-B, human corneal epithelial cell, photodamage, cytoprotective effects

## Abstract

Excessive UV-B exposure is well known to be a risk factor for corneal phototoxicity including direct DNA damage and disturbances in the antioxidant balance. Here, we showed a successful synthesis of a water-soluble and biocompatible small molecule **DHPM 1** with dihydropyrimidinthione skeleton, which could effectively protect human corneal epithelial (HCE-2) cells from UV-B damage. In separate experiments, **DHPM 1** absorbed UV-B rays and exhibited scavenging activity against intracellular ROS induced by UV-B radiation, thereby reducing the levels of DNA fragmentation. Additionally, UV-B exposure increased the expression of cleaved caspase-3, as well as the ratio of Bax/Bcl-2 at protein levels, while pretreatment with **DHPM 1** significantly reversed these changes. To the best of our knowledge, this is the first report of a study based on dihydropyrimidinthione derivatives to develop a promising eye drops, which may well find extensive applications in UV-B caused corneal damage.

## Introduction

Ultraviolet radiation (UV), an important risk factor for ocular diseases, is further categorised as: UV-C (200–280 nm), UV-B (280–320 nm) and UV-A (320–400 nm). Sunshine is a natural source of environment UV. As the shorter wavelengths of UV-C radiation are almost exclusively filtered by ozone in the stratosphere, the terrestrial environment is mainly exposed to UV-B (3%) and UV-A (97%) radiation (Ibrahim et al., 2012). Although it accounts for only ∼3% of terrestrial light, UV-B with a highly energetic wavelength is more biologically effective at damaging ocular tissue than UV-A. In addition, the human cornea absorbs ∼90% of UV-B radiation, in contrast to only ∼30% in the UV-A region (Young, 2006). Therefore, the eye (especially cornea) is the most susceptible organ to UV-B induced damage aside from skin (Kolozsvari et al., 2002). Under physiological conditions, the cornea is a transparent avascular tissue, which protects the lens and retina in the eyes against UV-B induced damaging effects by absorbing the majority of UV-B radiation. The typical UV-B induced corneal disorders contain photokeratitis, pterygium, climatic droplet keratopathy, damage to the epithelium, edema and apoptosis of corneal cells. At the cellular level, UV-B induced corneal phototoxicity could be caused by direct DNA damage, as well as by the generation of reactive oxygen species (ROS) and inflammatory cytokines (Tsai et al., 2012; Bigagli et al., 2017). ROS overproduction can elicit DNA mutations, lipid peroxidation and protein denaturation and induce pro-inflammatory cytokines, which plays an important role in promoting corneal inflammation (Bigagli et al., 2017). Meanwhile, UV-B by itself can also trigger the activation of NLRP3 inflammasomes and the secretion of IL-1β and/or IL-18 in human corneal epithelial (HCE) cells (Korhonen et al., 2020).

3,4-Dihydropyrimidine-2(*1H*)-ones and thiones (**DHPMs**) are a class of heterocyclic compounds, which have been intensively investigated mainly due to their diverse pharmacological properties, including calcium antagonists, α_1a_ adrenoreceptor antagonists, anticarcinogens, antibacterial, antiviral, antioxidants, etc. (Kappe, 2000; Pineiro et al., 2013) Recently, Tao et al. have prepared a series of polymers with **DHPMs** side chains. The optimized polymer **P (1)(4)-co-P(PEGMA)** with attractive antioxidant profiles showed much better UV-C resistant capability than superoxide dismutase (SOD). ∼100% L929 cells remained viable with 10 mg/ml of **P (1)(4)-co-P(PEGMA)**, suggesting its excellent cellular safety. After UV-C radiation (254 nm, 0.27 J/cm^2^), **P (1)(4)-co-P(PEGMA)** could protect cells from UV-C damage in a dose-dependent manner, especially almost 100% protection was achieved at 5 mg/ml (Mao et al., 2018). In order to further improve the UV protective abilities, three fluorescent polymers were prepared by introducing conjugated moieties into the original polymer structures and they were superior to the original polymer in effectively preventing UV-C induced DNA damage (Mao et al., 2021).

Encouraged by its excellent free-radical scavenging and UV resistant activities of **DHPMs**, we therefore explored designing a phosphotyrosine-containing small molecule of DHPMs as an alternative approach to achieve the excellent protection in the UV-B irradiated HCE-2 cell line. Phosphotyrosine [H-Try (H_2_PO_3_)-OH] with highly aqueous solubility at neutral pH can significantly improve the biocompatibility of **DHPMs** (Shy et al., 2020), which still require further refinement to achieve the applications in medicine. We have also explored the therapeutic potential of the new designed small molecule by investigating whether it can alleviate DNA damage and reduce ROS overproduction when administered before UV-B exposure.

## Materials and Methods

### Materials

Unless otherwise noted, solvents and reagents were obtained from commercial sources and used without further purification. Dulbecco’s modified eagle’s medium (DMEM, Gibco), phosphate buffered saline (PBS, Gibco), fetal bovine serum (FBS, Gibco), trypsin-EDTA (0.25%, Gibco), Alexa Fluor® 488-conjugated rabbit anti-phospho-histone H2A.X (Ser139) (20E3) mAb (Cell Signaling Technology, United States), Casepase 3 (active) rabbit monoclonal antibody (Beyotime Biotech, China), Bax rabbit monoclonal antibody (Beyotime Biotech, China), Bcl-2 rabbit monoclonal antibody (Sino Biological, China), β-actin rabbit monoclonal antibody (Beyotime Biotech, China), Calcein-AM/PI Double Stain Kit (Beyotime Biotech, China), Reactive Oxygen Species Assay Kit (Beyotime Biotech, China), Cell Counting Kit-8 (APExBIO Technology LLC, United States) were used as purchased.

### Instruments

High-resolution mass spectra (HRMS) were obtained on a Thermo Exactive Plus spectrometer. NMR spectra were recorded on Varian Mercury 400 MHz spectrometers. The purity of final products was determined by high-performance liquid chromatography (HPLC). The UV-B light source (NanJing Nationol Electronic Co. Ltd., China) was a large area irradiation ultraviolet lamp that emitted 106 μW/cm^2^ at the distance of 10 cm. The wavelength range of UV-B was 280–320 nm, with an average of 302 nm. UV absorption spectra were recorded on a UV-VIS spectrophotometer (UV 1800 SPC, Macy, China) using quartz cuvettes of 1 cm path length. The flow cytometry analyses were performed on a BD FACSCanto^TM^ flow cytometer (λex 488 nm and λem 500–600 nm for phospho-histone H2A.X (Ser139) (20E3) rabbit mAb). Confocal microscopic images were obtained on a Zeiss 780 using the following filters: λex 488 nm and λem 500–600 nm for DCF; λex 488 nm and λem 500–530 nm for Calcein AM; λex 561 nm and λem 600–700 nm for PI. The blots were visualized with Clarity Western ECL Substrate (Applygen, China) on Chemiluminescence imaging system (Tanon-5200 Multi, China).

## Methods

### Chemicals

Synthesis of compound **4**. Benzaldehyde (530 mg, 5.0 mmol), methyl acetoacetate (580 mg, 5.0 mmol), *N*-methylthiourea (675 mg, 7.5 mmol), acetic acid (5.0 ml) and magnesium chloride (95 mg, 1.0 mmol) were successively added to a 100 ml centrifuge tube. The tube was sealed and stirred for 2 h at 100°C. After completion, the reaction mixture was cooled and poured into crushed ice with vigorous stirring. The obtained crude was filtered, washed with cold water and diethyl ether to afford a white powder (1.2 g, 87%). HRMS calcd for C_14_H_17_N_2_O_2_S [M + H]^+^ 277.1005, found 277.1011; ^1^H NMR (400 MHz, DMSO-*d*_*6*_): δ 9.87 (d, *J* = 4.8 Hz, 1H), 7.36–7.18 (m, 5H), 5.21 (d, *J* = 4.8 Hz, 1H), 3.64 (s, 3H), 3.48 (s, 3H), 2.53 (s, 3H); ^13^C NMR (100 MHz, DMSO-*d*_*6*_): δ 150.4, 140.5, 126.5, 121.5, 110.8 (2C), 110.0, 108.7 (2C), 92.0, 49.6, 49.1, 36.8, 20.9.

Synthesis of compound **5**. To a solution of methanol (5 ml) and 1 N NaOH solution (aq., 10 ml) was added compound **4** (552 mg, 2.0 mmol) and then refluxed for 1 h. After cooled to room temperature, the reaction mixture was poured onto crushed ice, acidified with 1 N HCl (aq.). The crude product was filtered and dried to afford compound **5** (430 mg, 82%). HRMS calcd for C_14_H_17_N_2_O_2_S [M + H]^+^ 263.0849, found 263.0850; ^1^H NMR (400 MHz, DMSO-*d*_*6*_): δ 12.56 (s, 1H), 9.78 (d, *J* = 3.6 Hz, 1H), 7.36–7.31 (m, 2H), 7.28–7.20 (m, 3H), 5.21 (d, *J* = 3.6 Hz, 1H), 3.47 (s, 3H), 2.52 (s, 3H); ^13^C NMR (100 MHz, DMSO-*d*_*6*_): δ 178.1, 167.1, 147.4, 142.2, 128.6 (2C), 127.6, 126.0 (2C), 106.2, 52.3, 36.1, 16.2.

**DHPM 1** was synthesized by solid phase peptide synthesis (SPPS). 2-Chlorotrityl chloride resin (1.0 g, 1 mmol) was swelled in anhydrous dichloromethane for 20 min. Fmoc-L-Tyr (H_2_PO_3_)-OH (1.45 g, 3 mmol), DIEA (825 μL, 5 mmol) was dissolved in anhydrous DMF and then conjugated to swelled resin for 2 h. Subsequently washing with anhydrous DMF for three times, blocking unreacted sites of resin with DCM/MeOH/DIEA (80:15:5) for 20 min and re-washing with anhydrous DMF for five times. 20% Piperidine in DMF was used to remove the Fmoc group for 30 min and then washed successively with anhydrous DMF, methanol, cyclohexane and dichloromethane. Fmoc-Gly-OH (890 mg, 3 mmol) was activated with HBTU/DIEA in anhydrous DMF and then conjugated to above-mentioned resin for 2 h. 20% Piperidine in DMF was used to remove the Fmoc group for 30 min. Subsequently washing successively with anhydrous DMF, methanol, cyclohexane and dichloromethane. Then, compound **5** (780 mg, 3 mmol) was activated with HBTU/DIEA and conjugated to the resin for 2 h. Subsequently washing successively with anhydrous DMF, methanol, cyclohexane and dichloromethane. **DHPM 1** was cleaved off the resin with TFA for 2 h. After removing the solvent, anhydrous ether was added under sonication to afford the crude product, which was further purified by reversed-phase HPLC (420 mg, 75%). HRMS for C_24_H_28_N_4_O_8_PS [M + H]^+^: 563.1366; found 563.1357; ^1^H NMR (400 MHz, DMSO-*d*_*6*_): δ 9.48 (d, *J* = 4.8 Hz, 1H), 8.23 (dt, *J* = 5.6, 2.4 Hz, 1H), 8.19 (t, *J* = 7.2 Hz, 1H), 7.35–7.02 (m, 9H), 5.13 (d, *J* = 4.0 Hz, 1H), 4.45–4.37 (m, 1H), 3.92–3.75 (m, 1H), 3.73–3.56 (m, 1H), 3.41 (s, 3H), 2.22 (s, 3H), 3.05–2.95 (m, 1H), 2.90–2.78 (m, 1H); ^13^C NMR (100 MHz, DMSO-*d*_*6*_): δ 177.8, 172.7, 168.8, 166.8, 150.1, 142.1, 137.3, 132.8, 130.1 (2C), 128.5 (2C), 127.4, 126.0 (2C), 119.8, 119.7, 112.1, 53.6, 53.1, 41.8, 36.0, 35.8, 16.6.

### UV Absorption Spectroscopy

UV spectrum of **DHPM 1** (20 μg/ml) was performed with a UV-VIS spectrophotometer. The path length of the cuvette was 1 cm. The detection range was set to 200–400 nm and the spectral resolution to 1.0 nm.

### Cell Viability Assay

The human corneal epithelial cell line (HCE-2) was purchased from the American Type Culture Collection (ATCC, United States) and cultured in DMEM containing 10% FBS, 100 U/ml penicillin and 100 μg/ml streptomycin. Incubation was carried out at 37°C with a humidified atmosphere of 5% CO_2_. Cells in exponential growth phase were seeded in a 96 well plate at a concentration of 10^4^ cells/well and allowed to attach to the wells for 12 h. The culture medium was removed followed by addition of 100 µl culture medium containing different concentrations of **DHPM 1**. Parallel cultures of HCE-2 cells were irradiated with a UV-B lamp at the indicated dosages and then post-incubated for 24 h. Then, cell viability was detected by CCK-8 assay. All experiments were conducted triplicate. The results were calculated as means ± SD, which are expressed as cell viability (%).

### Cell Viability Imaging Assay

HCE-2 cells in exponential growth phase were seeded in a 35 mm glass-bottom dish (5 × 10^4^ cells) and allowed to attach to the dish for 12 h. The culture medium was removed followed by addition of 2.0 ml culture medium containing different concentrations of **DHPM 1**. Parallel cultures of HCE-2 cells were irradiated with a UV-B lamp at the indicated dosages and then post-incubated for 24 h. After washing with PBS, cells were further stained with commercial Calcein AM (2 μM) and PI (4.5 μM) at 37°C for 7 min in the dark. Cells were then washed twice with fresh live cell imaging solution and visualized by laser confocal microscopy (LSM 780, Carl Zeiss) immediately (λex: 488 nm for Calcein AM, 561 nm for PI; λem: 500–530 nm for Calcein AM, 600–700 nm for PI). The percentages of live/dead cells analysis was quantified with the fluorescence intensities of Calcein AM and PI, respectively.

### Intracellular ROS Assay

The cell samples were prepared as cell viability imaging assay and a fluorescence microscopic image analysis was performed using the ROS sensitive probe DCFH-DA as a tool for direct visualization of intracellular ROS generation in the HCE-2 cells. Briefly, **DHPM 1** (0 or 0.5 mg/ml) was added to the cells, which was irradiated or not irradiated with UV-B at 0.1 J/cm^2^. After incubation for 7 h, cells were washed with live cell imaging solution for three times, and further stained with commercial DCFH-DA (10 μM) at 37°C for 20 min in the dark. Cells were then washed two times with fresh live cell imaging solution and visualized by laser confocal microscopy (LSM 780, Carl Zeiss) immediately (λex: 488 nm, λem: 500–650 nm for DCF). The fluorescence intensity of DCF showing the generation of intracellular ROS level was analyzed by flow cytometer.

### DNA Damage Assay

HCE-2 cells in exponential growth phase were seeded in a 6 well plate at a concentration of 5 × 10^5^ cells/well and allowed to attach to the wells for 12 h. The culture medium was removed followed by addition of 2.0 ml culture medium containing different concentrations of **DHPM 1** (0 or 0.5 mg/ml). Parallel cultures of HCE-2 cells were irradiated or not irradiated with UV-B at 0.1 J/cm^2^. After incubation for 4 h, cells were harvested and then immobilized with 4% paraformaldehyde solution for 15 min followed by 90% methanol permeabilization for 10 min. The cells were washed with PBS and then kept in the Alexa Fluor 488-conjugated rabbit anti-phospho-histone H2A.X (Ser139) (20E3) mAb (1:50 dilution) solution for 1 h. Cells were washed with PBS and then analyzed by flow cytometer (λex: 488 nm, λem: 500–560 nm).

### Western Blot Analysis

HCE-2 cells in exponential growth phase were seeded in a 6 well plate at a concentration of 8 × 10^5^ cells/well and allowed to attach to the wells for 12 h. The culture medium was removed followed by addition of 2.0 ml culture medium containing different concentrations of **DHPM 1** (0 or 0.5 mg/ml). Parallel cultures of HCE-2 cells were irradiated or not irradiated with UV-B at 0.1 J/cm^2^. After incubation for 6 h, cultured cells were harvested and lysed in an RIPA buffer that contained an EDTA-free protease inhibitor cocktail. After centrifugation, the supernatants were retrieved and protein concentrations were measured with a BCA kit. Protein samples were separated by SDS-PAGE and transferred to PVDF membranes. After blocking with 5% skimmed milk, the membranes were incubated with various primary antibodies overnight at 4°C then incubated with secondary antibodies at room temperature for 1 h. The protein bands were detected with an enhanced chemiluminescence plus kit.

### Statistical Analysis

Statistical analysis was performed using GraphPad Prism Version 8.0 software. All the data are presented as the mean ± SD. Statistical difference was analyzed by One-Way ANOVA and *p* < 0.05 was considered statistically significant.

## Results

### Synthesis

**SCHEME 1:**
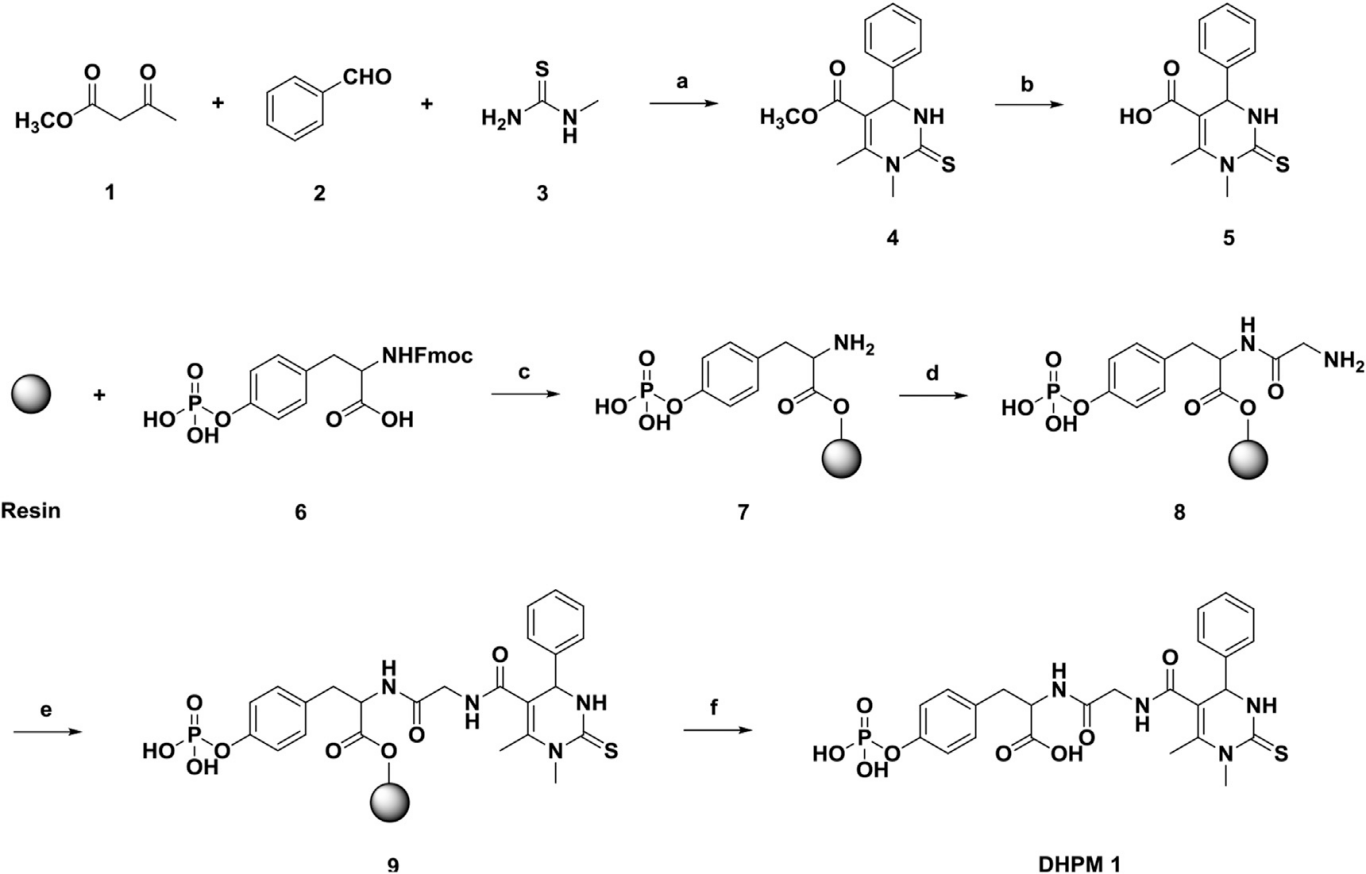
Reagents and conditions: **(a)** 20 mol% MgCl_2_, AcOH, 100°C; **(b)** NaOH; **(c)** 1) DIEA, DMF; 2) DCM/MeOH/DIEA (80:15:5); 3) 20% piperidine in DMF; **(d)** 1) Glycine, HBTU, DIEA, DMF; 2) 20% piperidine in DMF; **(e)** 7, HBTU, DIEA, DMF; **(f)** 95% TFA in CH_2_Cl_2_.

We began our exploration of water-soluble **DHPM 1** by applying tricomponent Biginelli reaction and solid phase peptide synthesis (SPPS). As shown in Scheme 1, methyl acetoacetate (**1**), benzaldehyde (**2**) and *N*-methylthiourea (**3**) were added to a tube. MgCl_2_ and acetic acid were used as the catalyst and solvent, respectively. The tube was sealed and then kept in a shaker at 100°C for 2 h. After completion, compound **4** was easily purified in 87% yield after simple washing with water and diethyl ether. Demethylation of **4** with 1 N NaOH (aq.) solution followed by acidification with diluted HCl afforded the desired compound **5** in high yield (82%). Subsequent transformation of **5** to the target **DHPM 1** was achieved employing Fmoc SPPS technique. Purification using preparative high-pressure liquid chromatography (HPLC) provided an 75% isolated yield of **DHPM 1** with a 98% purity. With the desired **DHPM 1** in hand, the water solubility was determined to be ∼2.5 mg/ml in PBS (pH 7.4).

### UV Absorption

**FIGURE 1:**
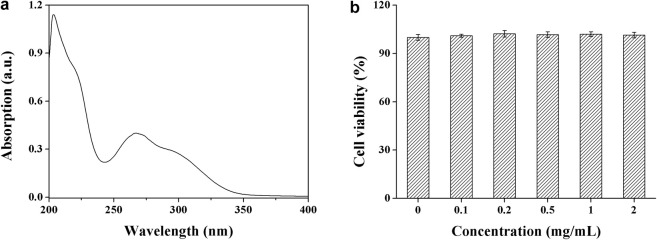
The UV absorption spectrum **(a)** and biocompatibility **(b)** of **DHPM 1**.

To test the cytoprotective ability of **DHPM 1** against the UV photodamage, we first checked the UV absorption spectrum ranging from 200 to 400 nm. As shown in Figure 1a, **DHPM 1** exhibited the strongest absorption at 206 nm, while there is no obvious absorption in the 350–400 nm region. In addition, **DHPM 1** showed an absorptive capacity for UV-B (280–320 nm) and the peak wavelength of absorption occurred at ∼300 nm. Thus, the UV (especially UV-B) absorbing properties of **DHPM 1** might be closely associated with its cytoprotective effect against UV-B radiation.

### Biocompatibility

Analysis of cell viability by CCK-8 assay revealed that there was no significant effect in response to 2 mg/ml **DHPM 1** in the non-irradiated cells during the 24 h incubation (Figure 1b), which indicated that **DHPM 1** possessed high biocompatibility with no observable *in vitro* toxicity and showed great promise for intracellular bio-applications.

### Cell Viability With UV-B Exposure

**FIGURE 2:**
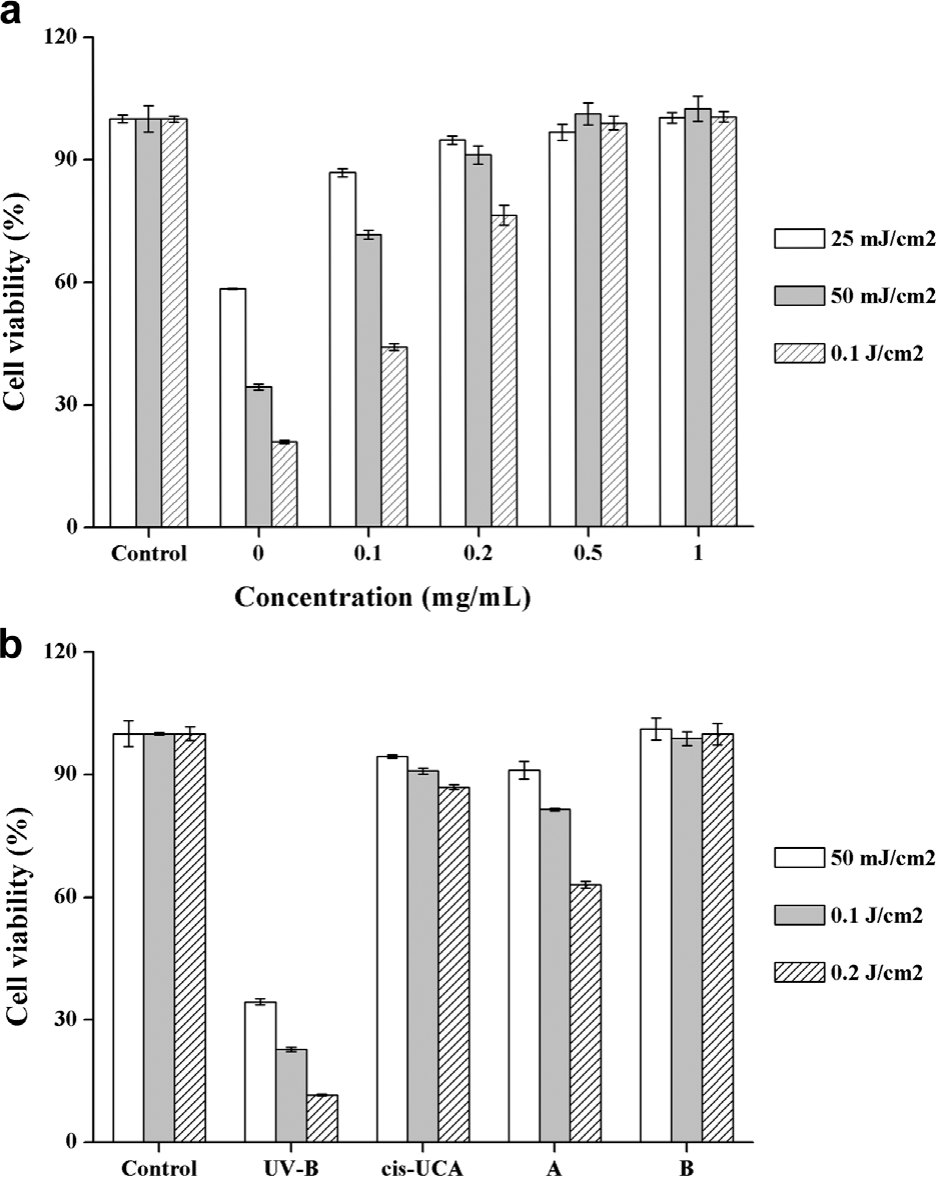
**(a)** Cell viability with **DHPM 1** at different concentrations after exposure to UV-B radiation (302 nm); **(b)** Cell viability with ***cis*-UCA** (0.1 mg/ml) and **DHPM 1** at different concentrations (A: 0.2 mg/ml; B: 0.5 mg/ml) after exposure to UV-B radiation (302 nm); non-irradiated cultures served as controls. The data were presented as means ± SD, *n* = 3.

To verify the cytoprotective effect of **DHPM 1** in UV-B irradiated HCE-2 cells, we examined its influence on cell survival. As shown in Figure 2a, cell viability in untreated control cells was 100%, whereas exposure of the HCE-2 cells to UV-B radiation induced a significant loss of viability in a dose-dependent manner, which were reduced to 58, 34 and 21%, respectively. On the contrary, 87, 72 and 44% cells remained viable with 0.1 mg/ml of **DHPM 1** and the cell viability was increased by 20–40% compared with corresponding UV-B radiation group in the absence of **DHPM 1**. Interestingly, **DHPM 1** at concentrations of 0.5 mg/ml and above could restore the metabolic activity of the UV-B irradiated cells to the level of the non-irradiated cells even at the high UV-B doses (0.1 J/cm^2^). These results suggested that **DHPM 1** could protect HCE-2 cells against UV-B radiation.

The most promising molecule reported in the literature so far is ***cis*-UCA** and 0.1 mg/ml ***cis*-UCA** was optimally anti-inflammatory and cytoprotective treatment option against UV-B induced inflammation and cellular damage in human corneal cells (Viiri et al., 2009; Korhonen et al., 2020). A head-to-head comparison of ***cis*-UCA** and **DHPM 1** demonstrated that cytoprotective effect of 0.2 mg/ml **DHPM 1** was equivalent to that of 0.1 mg/ml ***cis*-UCA** after UV-B exposure at an energy of 50 mJ/cm^2^. 0.2 mg/ml **DHPM 1** exhibited an accelerated reduction in cell viability by increasing doses of UV-B, 63% (**DHPM 1**) vs 87% (***cis*-UCA**) at 0.2 J/cm^2^ UV-B radiation. Encouragingly, 0.5 mg/ml **DHPM 1** could keep cell viability ∼100% even at the highest dose of UV-B radiation, which was better than that of 0.1 mg/ml ***cis*-UCA**. The molar concentrations of 0.5 mg/ml **DHPM 1** and 0.1 mg/ml ***cis*-UCA** are 890 and 724 μM, respectively, suggesting they have similar UV-B resistance capacities (Figure 2b).

**FIGURE 3:**
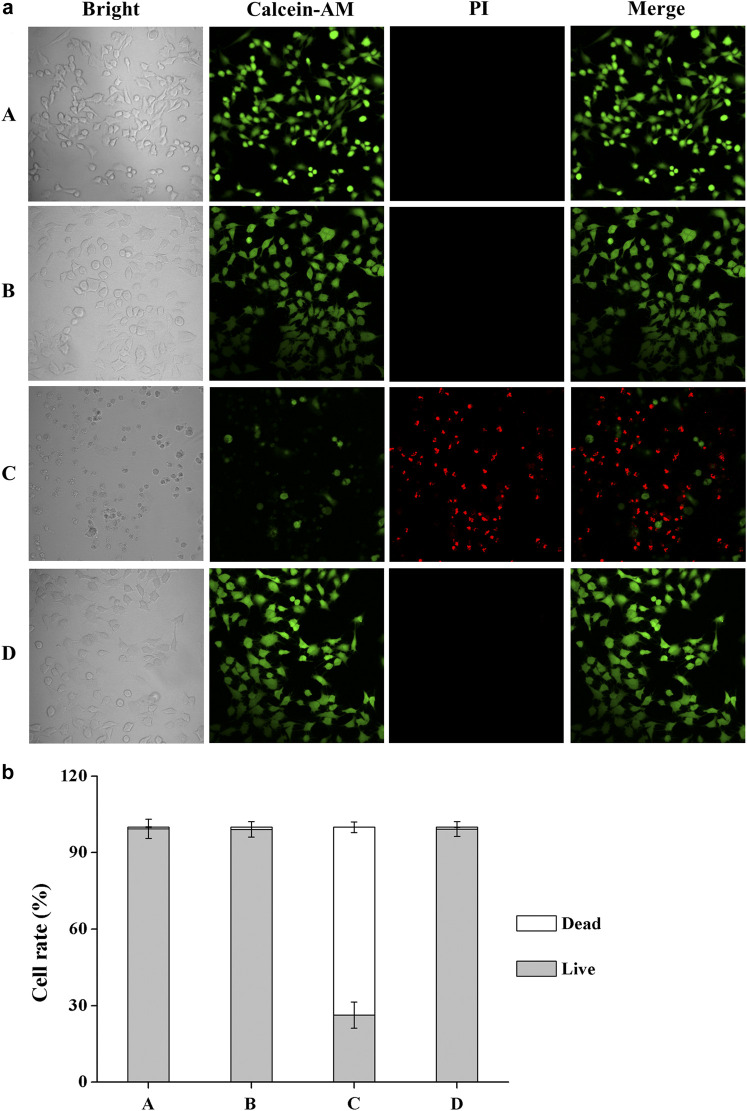
**(a)** Calcein AM/PI double staining of HCE-2 cells without UV-B radiation in the presence of **DHPM 1** at 0 **(A)** or 0.5 mg/ml **(B)**; with UV-B radiation in the presence of **DHPM 1** at 0 **(C)** or 0.5 mg/ml **(D)**; **(b)** The percentages of live/dead cells analysis in the groups **(A–D)** (*n* = 3).

The Calcein AM/PI assay is a rapid and simple approach to simultaneously observe living and dead cells (Calcein AM can enter and only accumulate in living cells, while PI only stains the nucleus of dead cells). We also used Calcein AM/PI double staining to qualitatively evaluate cell viability after UV-B radiation. After adding fresh culture medium containing **DHPM 1** (0 or 0.5 mg/ml), HCE-2 cells were exposed to UV-B radiation at the dose of 0.1 J/cm^2^ and then post-incubated for 24 h prior to further analysis. In the presence of **DHPM 1** (0 or 0.5 mg/ml), HCE-2 cells without UV radiation were tested as the control. Calcein AM/PI double staining revealed that most HCE-2 cells cultured without **DHPM 1** became shriveled and rounded, which were stained by PI as red spots after exposure to UV-B radiation. However, cells cultured with **DHPM 1** (0.5 mg/ml) survived the same process with viability similar to that of the control (Figure 3). These results agreed well with the quantitative data obtained by a CCK-8 assay (Figure 2), confirming that **DHPM 1** is a novel drug candidate to protect HCE-2 cells from fatal UV-B damage.

### ROS Generation

**FIGURE 4:**
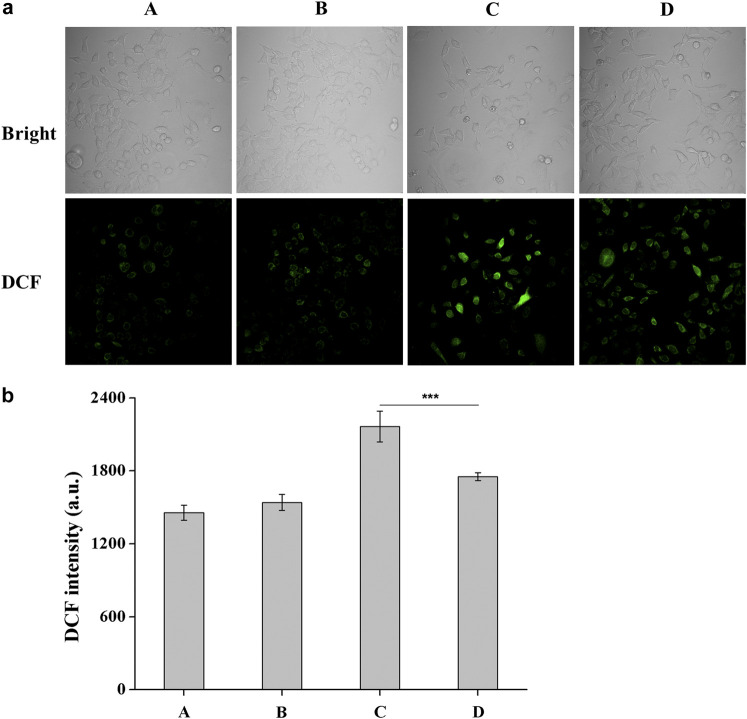
**(a)** Fluorescent images of DCFH-DA staining of HCE-2 cells without UV-B radiation in the presence of **DHPM 1** at 0 **(A)** or 0.5 mg/ml **(B)**; with UV-B radiation in the presence of **DHPM 1** at 0 **(C)** or 0.5 mg/ml **(D)**; **(b)** The DCF intensity analysis in the groups **(A–D)** (*n* = 3, ***p < 0.001).

DCFH-DA is an oxidation-sensitive fluorescent probe and can be oxidized to a highly fluorescent DCF (2′,7′-dichlorofluorescein), which corresponded to the increased ROS level in the HCE-2 cells. Combination with confocal microscope gives this method more simplicity and sensitivity for the observation of ROS generation. The microscopic images of DCF fluorescence showing the generation of intracellular ROS level were presented in Figure 4. The results obtained after 7 h of post-incubation revealed that UV-B exposure induced an obvious increase in the ROS level while a significant reduction in the DCF fluorescence was observed when **DHPM 1** (0.5 mg/ml) was applied, which indicated the intracellular ROS scavenging activities of **DHPM 1**.

### DNA Damage

**FIGURE 5:**
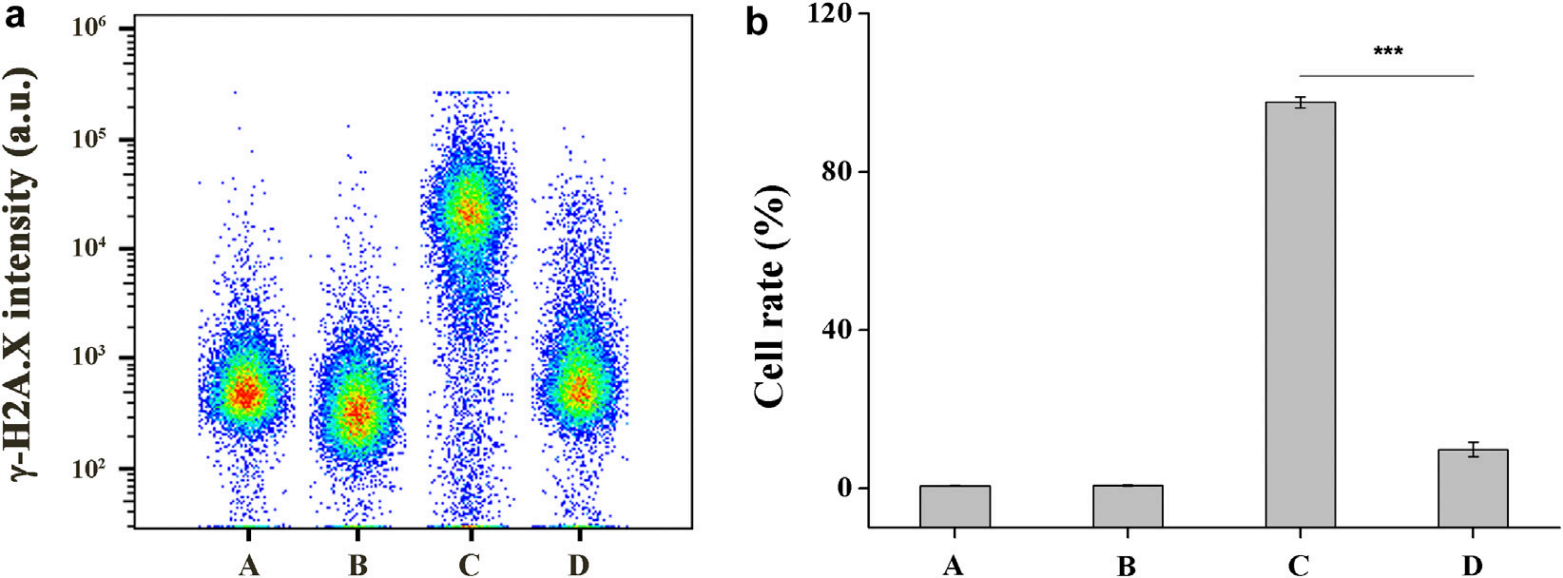
**(a)** Flow cytometry analysis of damaged DNA in HCE-2 cells without UV-B radiation in the presence of **DHPM 1** at 0 (A) or 0.5 mg/ml (B); with UV-B radiation in the presence of **DHPM 1** at 0 (C) or 0.5 mg/ml (D); **(b)** The percentages of DNA damaged cells analysis in the groups A–D (*n* = 3, ***p < 0.001).

UV-induced DNA damage can cause H2A.X to be rapidly phosphorylated by PI3K-like kinase at Ser139 site, which is a biomarker for evaluating DNA double-strand breaks (Paull et al., 2000). A flow cytometry-based quantification of phosphorylated H2A.X (γ-H2A.X) with Alexa Fluor 488 conjugated phospho-histone H2A.X (Ser139) (20E3) rabbit mAb was used to study the UV-B protection mechanism of **DHPM 1**. Flow cytometry analysis data indicated damaged DNA in 0.6 and 97.6% of cells before and after UV-B radiation, respectively. In contrast, the damaged DNA significantly decreased as the concentration of the **DHPM 1** increases from 0.1 to 0.5 mg/ml and reached up to 9.8% of cells cultured with 0.5 mg/ml of **DHPM 1** was detected after UV-B exposure, verifying that **DHPM 1** played an important role in protecting the UV-B induced DNA damage (Figure 5).

### Bax, Bcl-2 and cleaved Caspase-3 Expressions

**FIGURE 6:**
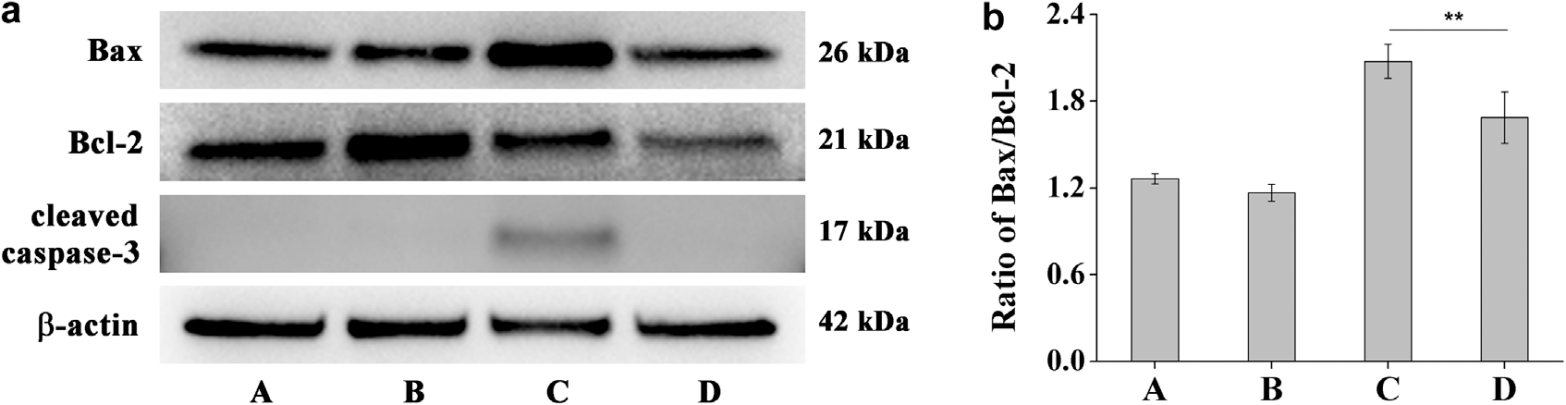
**(a)** Bax, Bcl-2 and cleaved Caspase-3 protein expression alterations in HCE-2 cells without UV-B radiation in the presence of **DHPM 1** at 0 (A) or 0.5 mg/ml (B); with UV-B radiation in the presence of **DHPM 1** at 0 (C) or 0.5 mg/ml (D); **(b)** The ratio of Bax/Bcl-2 protein expression in the groups A–D (*n* = 3, **p < 0.01).

The Bcl-2 and caspase families of proteins are related to the modulation of apoptosis process (Elmore, 2007). In order to better understand the protective mechanisms against UV-B induced apoptosis in HCE-2 cells, we analyzed the effect of **DHPM 1** on the expression of Bax, Bcl-2 and cleaved Caspase-3. Before exposure to UV-B irradiation, HCE-2 cells were pretreated with 0.5 mg/ml **DHPM 1**. The western blot results illustrated in Figure 6 showed that UV-B exposure significantly augmented the ratio of Bax/Bcl-2 at protein levels, which was restored by **DHPM 1**. Specifically, levels that were almost 1.6-fold and 1.3-fold higher than the control level were observed, respectively. In addition, UV-B exposure also stimulated the expression of cleaved Caspase-3, which was fully reversed in the presence of **DHPM 1**. Consistent with previous research, UV-B induced cell apoptosis through initiating the caspase-3 signaling pathway activation, which could be inhibited by **DHPM 1** to prevent the apoptosis of HCE-2 cells.

## Discussion

Skin and cornea are the two surfaces exposed to environmental UV radiation. Although human cornea shares many similarities with skin, there is a clear difference between corneal epithelial cells and keratinocytes. With uniquely specialized tissues, the cornea lacks features skin possesses, such as a thicker epithelial layer, stratum corneum and melanocytes, which help to resist UV damage. Therefore, the cornea on the anterior surface of the eye is particularly susceptible to the damage from excessive UV exposure, especially UV-B radiation (Roberts, 2001; Bashir et al., 2017). Clinically, acute UV-B exposure can cause photokeratitis, producing damage to the corneal epithelium, stroma and endothelium, and then develop into haze, edema and opacification. The sources of UV-B radiation include the well-known sunlight reflected off snow or off water and various high-intensity lamps. Photokeratitis is an inflammatory response and its clinical condition is generally reversible in most cases and largely self-resolving without any specific medical intervention. However, if the dose of UV-B exposure is substantial, inner corneal endothelial cells will be damaged and chronic UV-B exposure can also lead to climatic droplet keratopathy (CDK) and endothelial dysfunction. For humans, these damages would be expected to be irreversible (Doughty, 2019).

In the present work, we designed and synthesized a new dihydropyrimidinthione derivative (**DHPM 1**) with highly aqueous solubility and biocompatibility to ameliorate UV-B radiation mediated HCE-2 cell damage. Under different intensities of UV-B radiation (25–100 mJ/cm^2^), 0.1 mg/ml **DHPM 1** could increase the viabilities by 20–40%. More importantly, the viability of the HCE-2 cells was almost unaffected by 0.5 mg/ml **DHPM 1** treatment and its UV-B resistance capacity was equally to that of ***cis*-UCA**, a promising treatment option to suppress UV-B induced cellular damage in human corneal and conjunctival epithelial cells (Figures 2, 3).

UV-B can be directly absorbed by DNA, resulting in the formation of cyclobutane pyrimidine dimers (CPD) as well as pyrimidine 6-4 pyrimidone photoproducts (6-4 PP) followed by DNA damage or cell death (Bashir et al., 2017). In this work, **DHPM 1** can absorb UV-B energy and effectively inhibit HCE-2 cells damage. The absorption spectrum of **DHPM 1** overlapped with the UV-B spectrum (280–320 nm), suggesting that UV-B absorption effect of **DHPM 1** acts as a cytoprotective mechanism against UV-B induced cell damage (Tanito et al., 2003; Hyun et al., 2012). Additionally, an excessive UV-B exposure induces the production of ROS, such as hydrogen peroxide, singlet oxygen, hydroxyl radicals and superoxide anions, which can react with lipids, proteins and DNA, leading to the lipid peroxidation and, finally, cell death (Bashir et al., 2017). Indeed, several antioxidant agents, such as ascorbic acid, Fucoxanthin, Dacriovis™, EGCG, *Dunaliella salina* (*D. salina*), Rebamipide and Carteolol hydrochloride, have been reported to ameliorate UV-B induced corneal damage by reducing or preventing oxidative stress (Tanito et al., 2003; Suh et al., 2008; Chen et al., 2011; Tsai et al., 2012; Chen et al., 2014; Chen et al., 2016; Bigagli et al., 2017). As a kind of important antioxidant, **DHPMs** have also been intensively investigated as ROS scavenger (Pineiro et al., 2013). In the present study, we found that UV-B mediated oxidative damage caused an increase of intracellular ROS levels as compared to the normal control group, while treatment with **DHPM 1** significantly reversed these changes (Figure 4). Moreover, UV-B exposure can induce an inflammatory response in the cornea and inhibition of inflammatory factors has been considered as another treatment option for UVB-induced phototoxic status (Teng et al., 2018; Chen et al., 2020). In previous studies, ***cis*-UCA** was reported to be a promising anti-inflammatory compound, which could prevent the IL-1β and IL-18 secretion and therapeutically reduce the levels of IL-6, IL-8, and LDH in UV-B exposed HCE cells *in vitro* (Korhonen et al., 2020; Viiri et al., 2009; Jauhonen et al., 2011). This could serve as another way to investigate the protective mechanism of **DHPM 1** in UV-B mediated corneal damage.

The mechanisms of apoptosis are highly complex and sophisticated. There are two main apoptotic pathways: the extrinsic and intrinsic pathways. They converge on the same terminal, or execution pathway, which is initiated by the cleavage of caspase-3 and results in DNA fragmentation, etc. (Elmore, 2007) Previous studies have demonstrated that the intrinsic pathway is more important in UV-B induced apoptosis of corneal epithelial cells (Ubels et al., 2016; Du et al., 2017; Zhao et al., 2020; Maugeri et al., 2020). In our present study, UV-B induced an increase in the Bax/Bcl-2 ratio and the activation of caspase-3 in HCE-2 cells, which were prevented by **DHPM 1** to different extents (Figure 6). Thus, attenuating the intrinsic apoptosis pathway is the major mechanism underlying the protective effects of **DHPM 1** against UV-B induced HCE-2 cell damage.

## Conclusion

Although the antioxidant properties of **DHPMs** are well described in previous studies, this is the first work demonstrating that DHPMs prevents UVB-induced corneal damage *in vitro*. In the present work, we reported a new water-soluble dihydropyrimidinthione derivative **DHPM 1** with excellent cytoprotective properties against UV-B caused corneal damage. Our results demonstrated that the protective effects of **DHPM 1** likely derived from its ability to not only reduce the number of cell-damaging UV photons by the absorption spectrum but possibly attenuate ROS generation. **DHPM 1** also decreased the ratio of Bax/Bcl-2 and inhibited the activation of caspase 3, which subsequently prevented apoptosis via caspase 3 pathway. Topical **DHPM 1** eye drops may provide a safe and effective protective treatment option for UV-B induced damage on ocular surface. Therefore, further *in vivo* studies are required.

## Data Availability

The original contributions presented in the study are included in the article/Supplementary Material, further inquiries can be directed to the corresponding authors.

## References

[B1] Bashir H. Seykora J. T. Lee V. (2017). Invisible Shield: Review of the Corneal Epithelium as a Barrier to UV Radiation, Pathogens, and Other Environmental Stimuli. J. Ophthalmic Vis. Res. 12, 305–311. 10.4103/jovr.jovr_114_17 28791065 PMC5525501

[B2] Bigagli E. Cinci L. D'Ambrosio M. Luceri C. (2017). Pharmacological Activities of an Eye Drop Containing Matricaria Chamomilla and Euphrasia Officinalis Extracts in UVB-Induced Oxidative Stress and Inflammation of Human Corneal Cells. J. Photochem. Photobiol. B 173, 618–625. 10.1016/j.jphotobiol.2017.06.031 28704790

[B3] Chen B. Y. Lin D. P. Su K. C. Chen Y. L. Wu C. Y. Teng M. C. (2011). Dietary Zerumbone Prevents against Ultraviolet B-Induced Cataractogenesis in the Mouse. Mol. Vis. 17, 723–730. 21423870 PMC3060159

[B4] Chen M. H. Tsai C. F. Hsu Y. W. Lu F. J. (2014). Epigallocatechin Gallate Eye Drops Protect against Ultraviolet B-Induced Corneal Oxidative Damage in Mice. Mol. Vis. 20, 153–162. 24520184 PMC3919670

[B5] Chen S. J. Lee C. J. Lin T. B. Liu H. J. Huang S. Y. Chen J. Z. (2016). Inhibition of Ultraviolet B-Induced Expression of the Proinflammatory Cytokines TNF-α and VEGF in the Cornea by Fucoxanthin Treatment in a Rat Model. Mar. Drugs 14, 13. 10.3390/md14010013 26751458 PMC4728510

[B6] Chen W. Guo J. Guo H. Kong X. Bai J. Long P. (2020). Protective Effect of Vitamin C against Infancy Rat Corneal Injury Caused by Acute UVB Irradiation. Biomed. Res. Int. 2020, 8089273. 10.1155/2020/8089273 32596375 PMC7273459

[B7] Doughty M. J. (2019). Methods of Assessment of the Corneas of the Eyes Laboratory Rabbits Exposed to Solar Ultraviolet-B Radiation. Photochem. Photobiol. 95, 467–479. 10.1111/php.13031 30281803

[B8] Du S. Han B. Li K. Zhang X. Sha X. Gao L. (2017). *Lycium Barbarum* Polysaccharides Protect Rat Corneal Epithelial Cells against Ultraviolet B-Induced Apoptosis by Attenuating the Mitochondrial Pathway and Inhibiting JNK Phosphorylation. Biomed. Res. Int. 2017, 5806832. 10.1155/2017/5806832 28798932 PMC5536140

[B9] Elmore S. (2007). Apoptosis: a Review of Programmed Cell Death. Toxicol. Pathol. 35, 495–516. 10.1080/01926230701320337 17562483 PMC2117903

[B10] Hyun Y. J. Piao M. J. Zhang R. Choi Y. H. Chae S. Hyun J. W. (2012). Photo-protection by 3-bromo-4, 5-dihydroxybenzaldehyde against Ultraviolet B-Induced Oxidative Stress in Human Keratinocytes. Ecotoxicol Environ. Saf. 83, 71–78. 10.1016/j.ecoenv.2012.06.010 22795593

[B11] Ibrahim O. M. Kojima T. Wakamatsu T. H. Dogru M. Matsumoto Y. Ogawa Y. (2012). Corneal and Retinal Effects of Ultraviolet-B Exposure in a Soft Contact Lens Mouse Model. Invest. Ophthalmol. Vis. Sci. 53, 2403–2413. 10.1167/iovs.11-6863 22410564

[B12] Jauhonen H. M. Kauppinen A. Paimela T. Laihia J. K. Leino L. Salminen A. (2011). *Cis*-urocanic Acid Inhibits SAPK/JNK Signaling Pathway in UV-B Exposed Human Corneal Epithelial Cells *In Vitro* . Mol. Vis. 17, 2311–2317. 21921982 PMC3171492

[B13] Kappe C. O. (2000). Biologically Active Dihydropyrimidones of the Biginelli-Type-Aa Literature Survey. Eur. J. Med. Chem. 35, 1043–1052. 10.1016/S0223-5234(00)01189-2 11248403

[B14] Kolozsvári L. Nógrádi A. Hopp B. Bor Z. (2002). UV Absorbance of the Human Cornea in the 240- to 400-nm Range. Invest. Ophthalmol. Vis. Sci. 43, 2165–2168. 12091412

[B15] Korhonen E. Bisevac J. Hyttinen J. M. T. Piippo N. Hytti M. Kaarniranta K. (2020). UV-B-induced Inflammasome Activation Can Be Prevented by *Cis*-Urocanic Acid in Human Corneal Epithelial Cells. Invest. Ophthalmol. Vis. Sci. 61, 7. 10.1167/iovs.61.4.7 PMC7401861 32271889

[B16] Mao T. Liu G. Wu H. Wei Y. Gou Y. Wang J. (2018). High Throughput Preparation of UV-Protective Polymers from Essential Oil Extracts via the Biginelli Reaction. J. Am. Chem. Soc. 140, 6865–6872. 10.1021/jacs.8b01576 29627974

[B17] Mao T. He X. Liu G. Wei Y. Gou Y. Zhou X. (2021). Fluorescent Polymers via post-polymerization Modification of Biginelli-type Polymers for Cellular protection against UV Damage. Polym. Chem. 12, 852–857. 10.1039/D0PY00503G

[B18] Maugeri G. D'Amico A. G. Amenta A. Saccone S. Federico C. Reibaldi M. (2020). Protective Effect of PACAP against Ultraviolet B Radiation-Induced Human Corneal Endothelial Cell Injury. Neuropeptides 79, 101978. 10.1016/j.npep.2019.101978 31791645

[B19] Paull T. T. Rogakou E. P. Yamazaki V. Kirchgessner C. U. Gellert M. Bonner W. M. (2000). A Critical Role for Histone H2AX in Recruitment of Repair Factors to Nuclear Foci after DNA Damage. Curr. Biol. 10, 886–895. 10.1016/S0960-9822(00)00610-2 10959836

[B20] Pineiro M. Nascimento B. F. O. Rocha Gonsalves A. Md. A. (2013). Dihydropyrimidinone Derivatives: Redox Reactivity, Pharmacological Relevance and Medicinal applicationsIn: Quinones Occurrence, Medicinal and Physiological Importance. Editors Price E. R. Johnson S. C. (New York: Nova Science Publishers. Inc.), 1–44.

[B21] Roberts J. E. (2001). Ocular Phototoxicity. J. Photochem. Photobiol. B 64, 136–143. 10.1016/S1011-1344(01)00196-8 11744400

[B22] Shy A. N. Li J. Shi J. Zhou N. Xu B. (2020). Enzyme-instructed Self-Assembly of the Stereoisomers of Pentapeptides to Form Biocompatible Supramolecular Hydrogels. J. Drug Target. 28, 760–765. 10.1080/1061186X.2020.1797048 32668995 PMC7729926

[B23] Suh M. H. Kwon J. W. Wee W. R. Han Y. K. Kim J. H. Lee J. H. (2008). Protective Effect of Ascorbic Acid against Corneal Damage by Ultraviolet B Irradiation: a Pilot Study. Cornea 27, 916–922. 10.1097/ICO.0b013e31816f7068 18724154

[B24] Tanito M. Takanashi T. Kaidzu S. Yoshida Y. Ohira A. (2003). Cytoprotective Effects of Rebamipide and Carteolol Hydrochloride against Ultraviolet B-Induced Corneal Damage in Mice. Invest. Ophthalmol. Vis. Sci. 44, 2980–2985. 10.1167/iovs.02-1043 12824241

[B25] Teng M. C. Wu P. C. Lin S. P. Wu C. Y. Wang P. H. Chen C. T. (2018). Danshensu Decreases UVB-Induced Corneal Inflammation in an Experimental Mouse Model via Oral Administration. Curr. Eye Res. 43, 27–34. 10.1080/02713683.2017.1379543 29111819

[B26] Tsai C. F. Lu F. J. Hsu Y. W. (2012). Protective Effects of Dunaliella salina - a Carotenoids-Rich Alga - against Ultraviolet B-Induced Corneal Oxidative Damage in Mice. Mol. Vis. 18, 1540–1547. 22736944 PMC3380915

[B27] Ubels J. L. Glupker C. D. Schotanus M. P. Haarsma L. D. (2016). Involvement of the Extrinsic and Intrinsic Pathways in Ultraviolet B-Induced Apoptosis of Corneal Epithelial Cells. Exp. Eye Res. 145, 26–35. 10.1016/j.exer.2015.11.003 26559338 PMC4842139

[B28] Viiri J. Jauhonen H. M. Kauppinen A. Ryhänen T. Paimela T. Hyttinen J. (2009). Cis-urocanic Acid Suppresses UV-B-Induced Interleukin-6 and -8 Secretion and Cytotoxicity in Human Corneal and Conjunctival Epithelial Cells *In Vitro* . Mol. Vis. 15, 1799–1805. 19753313 PMC2742640

[B29] Young A. R. (2006). Acute Effects of UVR on Human Eyes and Skin. Prog. Biophys. Mol. Biol. 92, 80–85. 10.1016/j.pbiomolbio.2006.02.005 16600340

[B30] Zhao C. Li W. Duan H. Li Z. Jia Y. Zhang S. (2020). NAD+ Precursors Protect Corneal Endothelial Cells from UVB-Induced Apoptosis. Am. J. Physiol. Cel Physiol. 318, C796–C805. 10.1152/ajpcell.00445.2019 32049549

