# Supplementary material for: Cytoprotective Effects of Water Soluble Dihydropyrimidinthione Derivative Against UV-B Induced Human Corneal Epithelial Cell Photodamage
Source: Front Pharmacol. 2021 Oct 20;12:732833. doi: 10.3389/fphar.2021.732833 (PMC8570246; doi:10.3389/fphar.2021.732833)
Supplement: Supplementary file 1 [file DataSheet1.docx]

**Supporting Information**

Cytoprotective effects of water-soluble dihydropyrimidinthione derivative against UV-B induced human corneal epithelial cell photodamage

Enming Du^1^, Guojuan Pu^1^, Siyu He^1^, Fangyuan Qin^1^, Yange Wang^1^, Gang Wang^1^, Zongming Song^1^*, Junjie Zhang^1^*, Ye Tao^1,2^*

**^1^** Henan Eye Institute, Henan Eye Hospital, People's Hospital of Zhengzhou University, Henan University School of Medicine, Henan Provincial People's Hospital, Zhengzhou, China.

**^2^** Lab of Visual Cell Differentiation and Modulation, Basic Medical College, Zhengzhou University, Zhengzhou, China

***** Correspondence: Ye Tao ([toy1011@163.com](mailto:toy1011@163.com)); Junjie Zhang (zhangjj66@126.com); Zongming Song ([szmeyes@126.com](mailto:szmeyes@126.com)).

**S1:** The ^1^H-NMR spectrum of compound **4**

**S2:** The ^13^C-NMR spectrum of compound **4**

**S3:** The ^1^H-NMR spectrum of compound **5**

**S4:** The ^13^C-NMR spectrum of compound **5**

**S5:** The HPLC spectrum of **DHPM 1**

**S6:** The ^1^H-NMR spectrum of **DHPM 1**

**S7:** The ^13^C-NMR spectrum of **DHPM 1**

**S1:** The ^1^H-NMR spectrum of compound **4**

**S2:** The ^13^C-NMR spectrum of compound **4**

**S3:** The ^1^H-NMR spectrum of compound **5**

**S4:** The ^13^C-NMR spectrum of compound **5**

**S5:** The HPLC spectrum of **DHPM 1**

────────────────────────────

Rank Time Conc. Area Height

────────────────────────────

1 14.363 0.0360 5798 9288

2 14.527 98.2992 15842218 1143387

3 15.025 1.6648 268311 23921

────────────────────────────

Total 100 16116327 1176596

**S6:** The ^1^H-NMR spectrum of **DHPM 1**

**S7:** The ^13^C-NMR spectrum of **DHPM 1**
